# *In situ* pod growth rate reveals contrasting diurnal sensitivity to water deficit in *Phaseolus vulgaris*

**DOI:** 10.1093/jxb/erac097

**Published:** 2022-03-22

**Authors:** Andrew Merchant, Millicent R Smith, Carel W Windt

**Affiliations:** School of Life and Environmental Sciences, Faculty of Science, The University of Sydney, Sydney, NSW 2006, Australia; IBG-2: Plant Sciences, Forschungszentrum Jülich, Jülich, Germany; School of Life and Environmental Sciences, Faculty of Science, The University of Sydney, Sydney, NSW 2006, Australia; IBG-2: Plant Sciences, Forschungszentrum Jülich, Jülich, Germany; IBG-2: Plant Sciences, Forschungszentrum Jülich, Jülich, Germany; Northwest Agriculture and Forestry University, China

**Keywords:** Bean, drought, nuclear magnetic resonance, NMR, phenotyping, seed filling, yield

## Abstract

The development of reproductive tissues determines plant fecundity and yield. Loading of resources into the developing reproductive tissue is thought to be under the co-limiting effects of source and sink strength. The dynamics of this co-limitation are unknown, largely due to an inability to measure the flux of resources into a developing sink. Here we use nuclear magnetic resonance (NMR) sensors to measure sink strength by quantifying rates of pod dry matter accumulation (pod loading) in *Phaseolus vulgaris* at 13-min intervals across the diel period. Rates of pod loading showed contrasting variation across light and dark periods during the onset of water deficit. In addition, rates of pod loading appeared decoupled from net photosynthetic rates when adjusted to the plant scale. Combined, these observations illustrate that the rate of pod development varies under water limitation and that continuous, non-invasive methodologies to measure sink strength provide insight into the governing processes that determine the development of reproductive tissues.

## Teaser

Quantification of the rate of *Phaseolus*pod growth revealed contrasting impacts of water deficit during light and dark periods in pod-expansion and seed-filling phases of development.

## Introduction

The development of yield and its sensitivity to changes in growth conditions are the traditional focus of plant breeding programmes (Beebe *et al.*, 2008; Reynolds and Langridge, 2016). Recent reviews have emphasized improving rates of photosynthesis as a means to increase yield (Long *et al.*, 2006; Hogy *et al.*, 2010; Zhu *et al.*, 2010). More recently, the need to consider other factors that contribute to the development of sink and reproductive structures has been highlighted (Braun *et al.*, 2014; Körner, 2015; White *et al.*, 2016; Chang *et al.*, 2017; Smith *et al.*, 2018; Hageman *et al.*, 2020; Hageman and Van Volkenburgh, 2021). Whilst yield development is undoubtedly under multigenic control and influenced by a range of factors, numerous investigations have sought to characterize pre-determinates of yield for use in monitoring and selection programmes (e.g. Sulpice *et al.*, 2009). Despite these advances, an inability to measure real-time development of sink tissues has hampered efforts to couple physiological and chemical processes with their corresponding impact on yield.

Sink strength of reproductive tissues and its perceived impact on plant-scale processes have received significant consideration across many studies (for most recent review see White *et al.*, 2016). For plants, the determinacy of pod development is a critical property that underscores reproductive viability and, ultimately, survival. Coupling of pod development with changes in the environment or resource availability is largely unstudied due to the challenges in measuring pod development *in situ*. The impact of short-term environmental change or the onset of nutrient deficiency likely plays a significant role in the generation of the ‘yield gap’ of many crops (Lobell *et al.*, 2009; Sandana *et al.*, 2009; van Ittersum and Cassman, 2013; van Ittersum *et al.*, 2013). Addressing this gap between current and potential yields necessitates further interrogation of the coupling of pod (sink) development throughout reproductive ontogeny to determine the quantitative and qualitative sensitivity of development to more commonly phenotyped physiological processes in plant biology.

Plant size is an integrated measure of net growth rate over time. Similarly, seed or pod size is the integrated measure of growth rate across the developmental period of the reproductive tissue. Neither growth nor the development of reproductive tissues follows a linear growth rate. For reproductive tissues, patterns in tissue growth rate are influenced by a range of factors, including tissue heterogeneity (e.g. seed and pod development), resource availability (e.g. pulse water supply) and developmental processes (e.g. pod expansion and seed maturation). In addition, diel variation in light intensity requires ‘buffering’ of the photoassimilate supply to maintain growth and metabolism during dark periods (see for example Smith and Stitt, 2007; Stitt *et al.*, 2010; Graf *et al.*, 2010; Graf and Smith, 2011; Okamura *et al.*, 2021). The accumulation of starch in the chloroplast is a central mechanism by which this is achieved (Sulpice *et al.*, 2009; Stitt *et al.*, 2010). Similarly, short term changes in evaporative demand (usually expressed in terms of vapour pressure deficit) alter the requirement for water to be allocated to evaporative cooling and thus water can be allocated to mechanisms such as carboxylation or the maintenance of turgor pressure in actively growing tissues (for review see Tardieu *et al.*, 2014). Combined, these diel changes in environmental conditions likely impart changes in the capacity for synthesis and transport processes in plants to satisfy the demand for photosynthates by sink tissues. Despite their potential significance, such temporal variations in the filling rate of sink tissues such as pods and seeds of leguminous species have not yet been quantified. Non-invasive and non-destructive methodologies are required to quantify the rate of development. The dynamics of this relationship over diel time frames is not seen in calculations of final yield but may offer novel traits that can improve the resilience of yield production and develop a more comprehensive, mechanistic approach to addressing the yield gap.

In recent years nuclear magnetic resonance (NMR) and magnetic resonance imaging (MRI) based methods have enabled the monitoring and quantification of physiological and chemical processes in the intact plant, ranging from anatomical imaging to the physicochemical properties of plant organs (Van As, 2007), roots (van Dusschoten *et al.*, 2016), micro-imaging of seeds (Munz *et al.*, 2017), and xylem/phloem sap flow (Windt *et al.*, 2009; Van de Wal *et al.*, 2017). To date, the size, complexity, and fragility of NMR and MRI hardware have restricted the application of these techniques to specialized laboratories, limiting the characterization of plant function under tightly controlled, artificial conditions. The development of small-scale digital spectrometers, new permanent magnetic materials, and new magnet designs have made NMR technology increasingly portable (Zalesskiy *et al.*, 2014). This has led to several recent technology demonstrations of mobile MRI in the field (Kimura *et al.*, 2011; Jones *et al.*, 2012; Meixner *et al.*, 2021). Similarly, more straightforward, sensor-like applications of mobile NMR have been demonstrated by using small scale NMR devices (NMR sensors; NMRS) as online sensors to measure changes in water content in growing trees and pods (Rascher *et al.*, 2011; Lechthaler *et al.*, 2016). More recently, an NMRS coupled with relaxometric analysis was shown to enable the concurrent, real-time quantification of both solid and liquid matter *in situ* (Windt *et al.*, 2021). Using a custom designed C-shaped NMR magnet, NMR coil, and magnet housing, plant samples can be inserted into the NMRS to monitor the development of plant tissues through developmental stages with little to no impact on the tissue under investigation (see also: Windt and Blümler, 2015). This capacity gives unprecedented insight into the dynamics of tissue growth with potential to add significantly to our fundamental understanding of seed yield development in cropping systems. The NMRS enables the observation of diel variation in organ growth with high precision (Rascher *et al.*, 2011; Windt *et al.*, 2021) facilitating the observation of growth rates across diurnal time scales and developmental processes. Using this approach, it has been shown that dry matter accumulation in reproductive tissues of wheat continues under dark conditions (Windt *et al.*, 2021), suggesting that processes such as starch remobilization and phloem transport supply photoassimilates for tissue maintenance and growth. The dynamics of these processes in response to water deficit likely influence the determinacy and susceptibility of yield development to changes in resource supply.

*Phaseolus vulgaris* (common bean) is an important legume for human nutrition, particularly in Latin America and Africa, due to its relatively high protein content (Beebe *et al*., 2001, 2013; Broughton *et al.*, 2003; Rascher *et al.*, 2011; Smith *et al.*, 2019). We chose *P. vulgaris* for the purposes of this study due to both its importance for global human nutrition and its tissue and growth characteristics. Pods of *P. vulgaris* commonly exceed 10 cm in length and are susceptible to abortion under adverse environmental conditions. In addition, plant growth and the development of reproductive tissues is ‘semi-determinant’ indicating strong coupling of development to environmental cues. Combined, these characteristics present challenges for predicting its sensitivity to changes in environmental conditions and ultimately, predicting yield (see Hageman *et al.*, 2020; Hageman and Van Volkenburgh, 2021). Considering these challenges, this study employs mobile NMRSs to concurrently investigate diel variations in pod development during the onset of water deficit in *P. vulgaris.* In particular, we address the following hypotheses to investigate the short term influence of water deficit on pod development *in situ*: (i) short term water limitation will impart a reduction in net photosynthetic rate and stomatal conductance, thereby reducing ‘source’ strength for photoassimilates; (ii) water deficit will impart a net reduction in the rate of pod growth over the diel cycle; (iii) the magnitude of reductions in pod growth will be equal under light and dark conditions; and (iv) the calculated magnitude of pod dry matter accumulation across the plant (sink) will scale proportionally with whole plant photosynthetic (source) rate.

## Material and methods

### Experimental design

Seeds of *Phaseolus vulgaris* were germinated in spring (April) and raised under glasshouse conditions at the Forschungszentrum in Jülich, Germany. A single genotype was used, ‘DOR390’, obtained from the International Centre for Tropical Agriculture breeding programme (CIAT, Colombia) (syn: Tuc390 (Argentina), Negro Tacana (Mexico)). DOR390 is a commercial cultivar that produces reliable yield under moderate stress. Details can be found at: https://genebank.ciat.cgiar.org/genebank (accession number G51400). Glasshouse conditions were set at 25/15 °C with relative humidity maintained between 60% and 80%. Light within the chamber fluctuated between 300 and 1200 μmol m^−2^ s^−1^ according to daily weather conditions and were supplemented to maintain the lower limit. Supplemented light arrays occupied a 145-degree angle of illumination in all directions from the leaf surface. Plants were sown into a commercial potting soil consisting of a peat base with commercial grade macro- and micronutrients added (ED 73, Stangenberg GmbH, Germany) in 8-litre pots. On each day, plants were watered to the field capacity of the soil by drip irrigation. By the time of treatment application, individual plants were 45 d old, approximately 600 mm in height, and the stems were approximately 2 cm in diameter.

Forty-four plants were allocated to each of a control treatment (full water supply) and a water deficit treatment. Water deficit treatments were applied to 8-week-old plants and therefore were in effect 15 d prior to the first NMR measurement. Water deficit was imparted by a controlled supply of water as a proportion of water used by control plants, also taking into account evaporation from the soil. Water deficit-treated plants received 30% of the water transpired by control plants. Loss of water due to evaporation from the soil surface was accounted for by incorporation into the calculation the weights of pots containing only the soil mixture at each watering event. Pots were arranged randomly and rotated weekly to avoid bench effects. Six plants in each treatment were set aside for predawn water potential (Ψ_pdwn_) measurements prior to the commencement of NMR measurements. Separate plants were used to measure Ψ to avoid changes in leaf area due to destructive sampling.

### Measurement of leaf area, pod weight, and pod length

At the end of the experiment, all 32 plants subjected to the NMRS were measured for total leaf area, total leaf dry weight, total pod weight (combined for one plant), pod number, and individual pod length. Leaf area was measured using a LI-COR LI-3100 leaf area meter (LI-COR, Lincoln, NE, USA). For pod length, individual pods were measured using a digital vernier caliper (Sontax, Perth, Australia). Individual pod lengths were then summed for each plant. After leaf area and pod length were measured, above-ground plant component (leaves, stems, pod) samples were dried in paper bags at 70 °C for 48 h then weighed. A randomized sub-sample of pods were taken from five plants in each treatment and measured for individual pod length and individual pod weight to enable allometric calculation of pod size based upon pod segment weight in the NMRS.

### Leaf gas exchange, predawn leaf water potential, and carbon isotope abundance measurements

Leaf gas exchange was measured on each plant using a LI-COR 6400 XT infra-red gas analyser for leaf-level photosynthesis from 08.00 to 17.00 h on the day of NMR measurement. Each plant was measured sequentially six times across the light period with a different fully expanded, non-shaded leaf chosen for measurement at each time point. Light conditions, temperature, and CO_2_ mole fraction of reference air were set to tracking mode while temperature and relative humidity in the measuring chamber set/maintained to prevailing growth conditions. Net CO_2_ assimilation rate (*A*, μmol m^−2^ s^−1^), stomatal conductance to water vapour (*g*_s_, mmol m^−2^ s^−1^) and sub-stomatal CO_2_ concentration (*c*_i_, μmol mol^−1^) were logged and then averaged across the day. Using a separate set of 12 plants (six per treatment) subjected to the same watering regime, predawn leaf water potential was measured 2 d prior to the commencement of NMR measurements using a Scholander pressure chamber (PMS, Corvallis, OR, USA).

For carbon isotope abundance, fully expanded leaf material was collected at midday on each of the sampling days. Ten first fully expanded leaves were collected from the canopy, with leaves taken from several heights. Leaves were immediately microwaved as per Popp *et al.* (1996) and then oven-dried at 75 °C. Samples were then ground to a powder and kept at −80 °C awaiting analysis. For the analysis of carbon isotope composition of the soluble extract in leaves (δ^13^C_sol_), 40 mg of ground leaf material was weighed into a 2 ml microtube to which 1 ml of hot, deionized water was added and incubated for 1 h at 75 °C. Samples were centrifuged at 11 400 *g* for 3 min and 800 μl of the supernatant transferred to a 2 ml micro-tube. Two hundred microlitres of soluble extract was then transferred into tin cups and dried at 45 °C in preparation for analysis. Samples were analysed using isotope ratio mass spectrometry on an Isochrom mass spectrometer (Micromass, Manchester, UK) coupled to a Carlo Erba elemental analyser (CE Instruments, Milan). Samples were dropped from an AS200 auto-sampler and combusted by Dumas combustion in a furnace kept at 1060 °C. Carbon isotope ratios are expressed in delta-notation, where δ^13^C=*R*_sample_/*R*_standard−_1, and *R* is the ratio of ^13^C to ^12^C in a sample and standard (Vienna Pee Dee Belemnite), respectively.

### Measurement of pod growth using nuclear magnetic resonance

Proton density of pod segments was measured using a set of four NMR plant sensors, using the solid and liquid content (SLC) method outlined by Windt *et al.* (2021). In brief, in the SLC method the proton density (PD) of protons in liquids (PD_liq_) and in solids (PD_sol_) is determined on the basis of their NMR relaxometric properties. The NMR signal of protons in solids decays very rapidly, with T_2_ (or spin–spin) relaxation times in the order of microseconds, whereas the signal of protons in semisolids and liquids relaxes much more slowly (milliseconds to seconds). The total proton density (PD_tot_) is determined on the basis of an extrapolation of the relaxation curve of the free induction decay (FID). By means of the FID the fastest as well as the slower relaxing protons are measured. A measure of the proton density of the liquid fraction (PD_liq_) was determined on the basis of the Carr–Purcell–Meiboom–Gill (CPMG) sequence, taking the average signal intensity of all data points on the relaxation curve between 0 and 25 ms. PD_tot_ correlates linearly with fresh weight of the sample, PD_liq_ with water content of the sample, and the difference between the two, solid proton density (PD_sol_=PD_tot_−PD_liq_), with dry weight of the sample (for details, see Windt *et al.*, 2021).

Each NMRS consisted of a C-shaped permanent magnet with a field strength of 0.234 T (10 MHz), an air gap of 37 mm and a pole diameter of 75 mm. The magnets were fitted with a probe holder that could be rotated to fit the angle of the sample (Windt *et al.*, 2021). To allow light to reach the sample, the probe holders were half open; entry of radio frequency (RF) noise was prevented by completing the Faraday cage that consists of the assembly of probe holder and magnet with a thin silver mesh. The NMR RF coil, positioned in the isocentre of the NMR magnet, defines the place and dimensions of the spot where the measurement is made. The solenoidal RF coil employed here had an inner diameter of 20 mm, a length of 30 mm, and 13 windings of 0.4 mm copper wire, wound on a glass carrier mould to allow light to reach the sample while in the sensor. The NMRSs were driven with a standard Kea II spectrometer (Magritek, Wellington, New Zealand), equipped with the standard, built-in 100 W RF amplifier. The spectrometer was mounted in a temperature-controlled housing and run at a constant temperature of 35 °C. All measurements were made on the basis of a free induction decay (FID)–CPMG sequence with the following parameters: 90 degree pulse 4 µs and −3 dB; 180 degree pulse 16 µs and −6.5 dB; spectral width 2 MHz, dead time 10 µs; echo time 350 µs, 6000 echoes, 64 repetitions, and a repetition time of 7.5 s. Data evaluation was performed as described by Windt *et al.* (2021).

Pods were placed into the NMRS by positioning the pod to hang by the petiole from the top of the coil housing. Pods were secured by loose-fitting foam, sitting within the glass coil former but outside the measurement field. Approximately 2.5 cm of each pod was within the coil, hence constituting the measured tissue. The NMR coil employed in this study spanned the circumference of the pod (coil diameter=20 mm) but only a segment of the length (coil height=30 mm). Consequently a standardization procedure was employed to calculate the relative increase in dry matter according to pod size using the formula:


$$\begin{array}{l} \Delta {\left(PD_{sol}\right)}_{w}=\;\left({\left(PD_{sol}\right)}_{T_{N}}-{\left(PD_{sol}\right)}_{T_{0}}\right)/{\left(PD_{sol}\right)}_{T_{0}} \end{array}$$ (1)

where (PD_sol_) represents the proton density determined by NMR-based relaxometry at the beginning of the measurement period (*T*_0_) and at time *N* (*T*_*N*_) and thus Δ(PD_sol_)_w_ equals the change in solid matter proton density compared with the initial observation weighted by the initial proton density measurement.

### Calibration of pod dry weight to proton density of the solid fraction with the NMRS

**Fig. 1.**
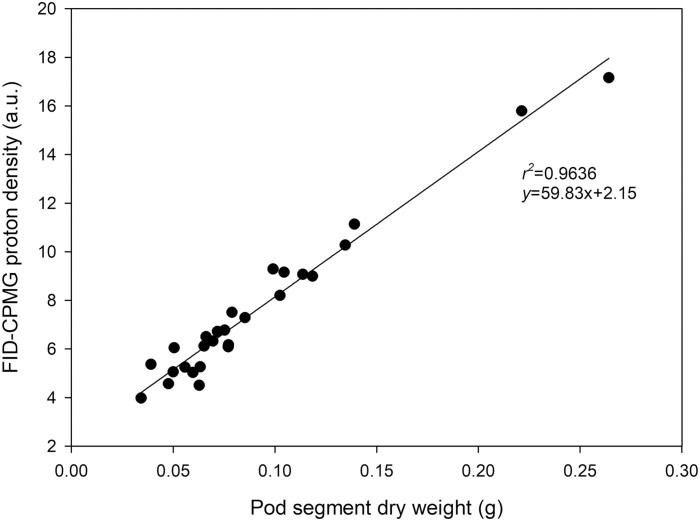
Proton density (a.u.) of the solid fraction (PD_sol_) of *P. vulgaris* beans plotted against pod segment dry weight measured gravimetrically. Samples were taken from a range of pod developmental stages from 6 to 25 d after pod set. Plants were watered daily to field capacity. Greenhouse conditions were set at 25/15 °C with a relative humidity maintained between 60% and 80%. Light conditions within the chamber fluctuated between 300 and 1200 μmol m^−2^ s^−1^.

Calibration of pod segment dry weight determined gravimetrically gave a significant (*P*<0.05) correlation with pod segment dry weight determined by the NMRS (Fig. 1).

### Development of ‘short term’ application of the NMRS to determine relative pod growth

**Fig. 2.**
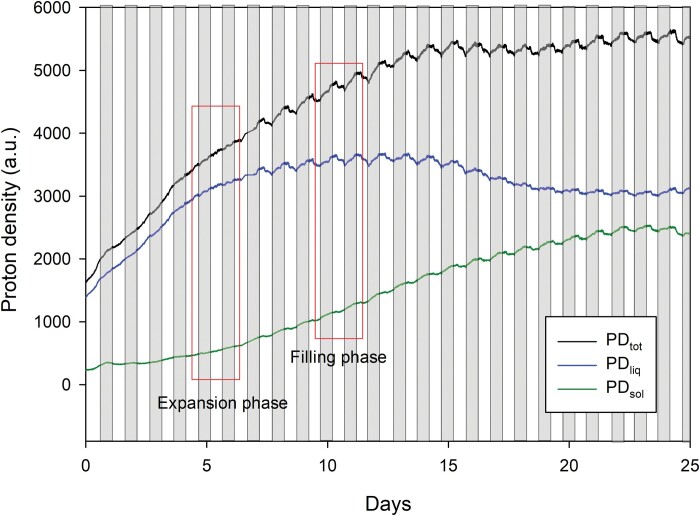
NMRS quantification of total proton density (PD_tot_), total liquid proton density (PD_liq_), and total solid matter proton density (PD_sol_) of a single pod of *Phaseolus vulgaris* across a 27 d period, encompassing both expansion and filling phases. Greenhouse conditions were set at 25/15 °C with a relative humidity maintained between 60% and 80%, and the plant was watered daily to field capacity. Light conditions within the chamber fluctuated between 300 and 1200 μmol m^−2^ s^−1^.

**Fig. 3.**
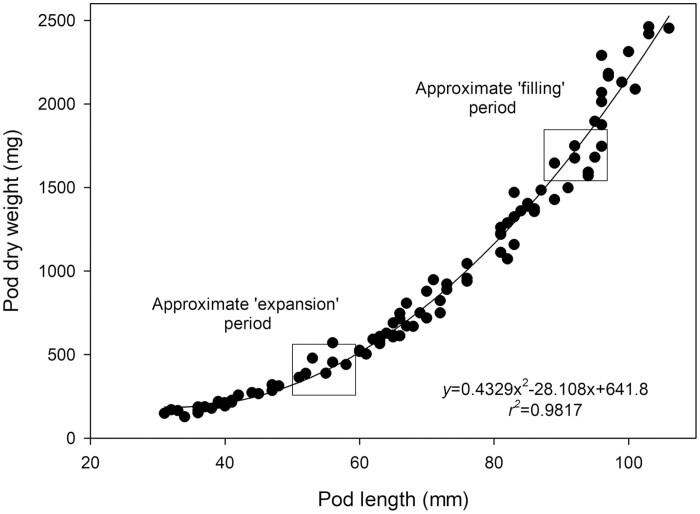
Pod length versus pod weight taken from 10 plants of *Phaseolus vulgaris*. Plants were watered daily to field capacity. Greenhouse conditions were set at 25/15 °C with a relative humidity maintained between 60% and 80%. Light conditions within the chamber fluctuated between 300 and 1200 μmol m^−2^ s^−1^.

So far, NMRS measurements have only been described as completed for the duration of pod or spike development. To facilitate shorter term, comparable measurements on larger numbers of plants using NMRSs, we stratified our study to focus on comparisons between pod developmental phases. Pod selection, the timing of measurement, and standardization of the NMRS measurement in both time and space were critical for the purposes of this study. Proton density of a pod segment follows a multiphase pattern that correlates with observations by Tanaka and Fujita (1979) showing an initial expansion phase, a filling phase, and finally a reduction in PD_sol_ that is thought to signify the moment that the hull of the pod senesces. This development also becomes noticeable in the dramatic drop in PD_liq_ in the last days, which potentially may also be associated with the time point from which part of the solid matter in the seeds turns crystalline. In the configuration used in this study, crystalline solids become invisible to the NMRS (Windt *et al.*, 2021). To illustrate this pattern we measured proton density over the course of pod development for a single pod (Fig. 2). To account for transition between expansion and filling phases, a relationship was also established between total pod length and its corresponding weight by harvesting 60 bean pods of differing age (taken from separate plants). The timing of subsequent short term (36 h) NMRS measurements was then set according to the pod being within the ‘expansion’ or ‘filling’ stage of development (Figs 2, 3).

Absolute accumulations of proton density measured for each individual bean pod were weighted according to developmental stage based on the individual pod length taken at the end of the measurement period. Total pod wet and dry matter were determined gravimetrically as well as the dry weight of the pod segment that was located within the NMR coil, the accuracy of which was made possible due to the ‘open’ C-shaped NMR design and the glass carrier mould of RF coil.

Flowering and pod initiation was closely monitored with individual flowers/pods tagged according to age. Twelve days following pod set, two plants from each treatment were allocated to four NMRSs. Pods were loaded into the NMR coil and stabilized using soft foam to prevent movement during the measurement period. Pod segments were measured for a 36 h period. To reduce the effects of external RF noise produced by adjacent electrical machines in the greenhouse, pod incremental data are presented over time using a 3-point moving average function.

### Estimations of total ‘source’ and sink ‘strength’

Estimates of total source and sink ‘strength’ were calculated by scaling both net photosynthetic rate (source) and pod loading rate (sink) to the plant scale. For plant-scale net photosynthetic rate, net photosynthesis measured using an IRGA (μmol CO_2_ m^−2^ s^−1^) during the NMR measurement period was scaled to the total leaf area of the plant (source strength, μmol CO_2_ s^−1^). Based upon preliminary tests, intra-canopy light conditions and maximum rates of photosynthesis (*A*_max_) values were assumed to be consistent. Plant-scale sink strength was determined by correlating individual pod lengths of all pods on 10 separate plants with dry weight (see Fig. 3) and calculating the rate of change in dry matter for each individual pod then summing to the plant scale. Specifically, pod length was converted to pod weight using the curvilinear relationship established between pod weight and pod length determined for this genotype separately (Fig. 3). The rate of change for each pod was then determined as a measure of loading rate (Eq. 2, which is the first derivative of the regression in Fig. 3) and summed for all pods on each plant.


$$\begin{array}{l} y\;=\;0.8658x\;-28.108 \end{array}$$ (2)

### Statistical analysis

Effects of treatments were analysed by analysis of variance using ‘Statistica’ analytical software (Version 6, StatSoft, Tulsa, OK, USA). *P*-values were calculated using Tukey’s honestly significant difference *post hoc* test mean values. Linear regressions were calculated using a general linear model. Differences in rates of dry matter accumulation were detected using *post hoc* testing on average rates of accumulation taken across the treatment period.

## Results

### Leaf gas exchange, predawn leaf water potential, and isotope abundance illustrated an isohydric response

Leaf gas exchange, averaged for each treatment revealed the impact of the water deficit treatment (Table 1). Reductions in both average net photosynthesis (*A*) and stomatal conductance (*g*_s_) were observed in response to the water deficit (*P*<0.05). Whilst an increase in average sub-stomatal carbon concentration was observed in water deficit treatment plants, this did not significantly differ between treatments (*P*>0.05). Similarly, Ψ_pdwn_ did not significantly differ between well-watered and water deficit treatments measured on adjacent plants, being 0.17 ± 0.12 MPa and 0.38 ± 0.15 MPa, respectively (mean ±SE). Subsequently, water deficit did not significantly influence the ratio between the ^13^C and ^12^C carbon isotopes (δ^13^C, *P*>0.05). Similarly, no gas exchange parameter (*A*, *g*_s_, or *c*_i_) correlated significantly with carbon isotope ratio (δ^13^C, Table 1)

**Table 1.** Mean leaf net photosynthetic rate (*A*), stomatal conductance, sub-stomatal CO_2_ concentration (*c*_i_), and carbon isotope abundance (δ^13^C) of *P. vulgaris* plants grown under controlled conditions and well watered (daily field capacity of the soil) or subject to water deficit (30% of transpiration of control plants)

	*A* (mmol m^−2^ s^−1^)	Stomatal conductance (mmol m^−2^ s^−1^)	*c*_i_ (ppm)	δ^13^C (‰)
Well watered	6.316 ± 0.564 (a)	0.180 ± 0.014 (a)	298.62 ± 7.93 (a)	−29.04 ± 0.22 (a)
Water deficit	5.863 ± 0.523 (b)	0.139 ± 0.025 (b)	311.27 ± 15.34 (a)	−29.31 ± 1.73 (a)
Slope	−0.1758	−0.0124	−12.057	NA
Intercept	0.949	−0.1878	−41.274	NA
*r* ^2^	0.004	0.001	0.022	NA
*P*-value	0.724	0.877	0.792	NA

Results are presented together with regression analysis of gas exchange parameters against measured δ^13^C. Gas exchange was measured at least six times over the course of the photoperiod. NA, not applicable. *Post hoc* groupings indicated by lowercase letters represent significant differences between treatments (Tukey HSD).

### Biomass partitioning was resilient to the imposition of water deficit

The imposition of water deficit did not influence leaf or stem dry weight, pod dry weight, total plant leaf area, or total plant pod length (*P*>0.05, Table 2). Consequently, photosynthetic rate expressed on the basis of whole plant leaf area significantly differed (*P*=0.04) between treatments with well-watered plants assimilating more carbon (41.45 ± 0.92 μmol plant^−1^ h^−1^) compared with water deficit-treated plants (38.85 ± 1.11 μmol plant^−1^ h^−1^). Stomatal conductance also significantly differed (*P*=0.02) among treatments with water deficit-treated plants exhibiting significantly lower values (0.921 ± 0.27 mmol plant^−1^ h^−1^) compared with well-watered plants (1.181 ± 0.19 mmol plant^−1^ h^−1^).

**Table 2.** Mean leaf area, leaf weight, total pod weight, and length (per plant) for 32 *Phaseolus vulgaris* plants that were well watered (16 plants) or subject to water deficit for a period of 21 d.

	Leaf dry weight (g)	Stem dry weight (g)	Pod dry weight (g plant^−1^)	Leaf area (cm^−2^ plant^−1^)	Pod length (mm plant^−1^)
Well watered	0.811 ± 0.072	11.08 ± 1.45	5.83 ± 0.89	1823 ± 187	1769 ± 152
Water deficit	0.883 ± 0.071	13.37 ± 1.70	5.14 ± 1.23	1841 ± 222	1688 ± 202
*P*-value	0.327	0.544	0.693	0.390	0.873

**Fig. 4.**
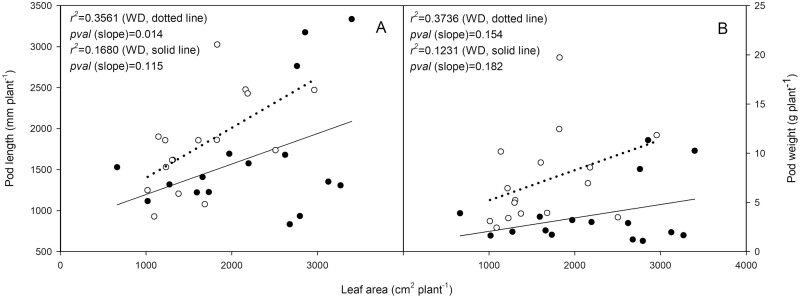
Total plant leaf area plotted against total plant pod length (A) and total plant pod dry weight (B) for 32 *Phaseolus vulgaris* plants that were well watered (*n*=16) or subject to water deficit for a period of 15 d. Filled symbols represent well-watered (control) treatment and open symbols represent water deficit treatment.

Total pod dry weight and length did not significantly scale with total leaf area nor with treatment. Whilst a significant positive slope was detected between total plant pod length and leaf area, the regression was weak (Fig. 4A). A similarly weak regression was observed between total plant leaf area and total pod weight (Fig. 4B) with a positive yet non-significant slope.

### Pod expansion phase: pod loading was impacted by water deficit only during the dark period

**Fig. 5.**
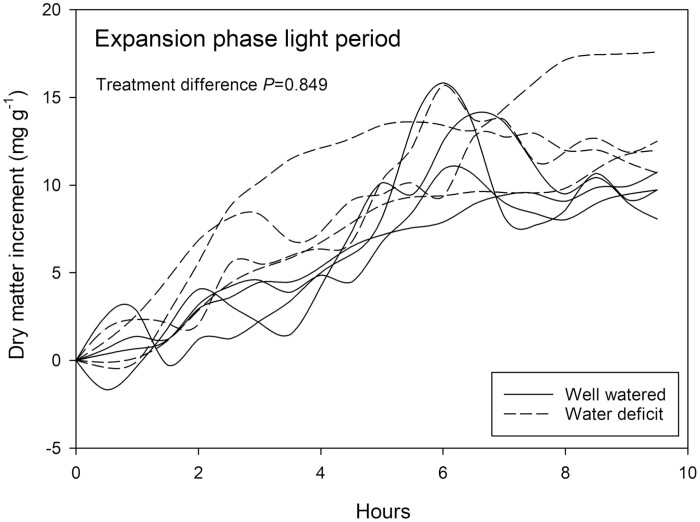
Relative change in solid matter (by weight) of *Phaseolus vulgaris* pod segments measured by NMRS for a 10 h period in the light during the pod expansion phase. Plants watered daily to field capacity (solid lines) and plants supplied with 30% of calculated water use of control plants are represented (dashed lines). Greenhouse conditions were set at 25/15 °C with a relative humidity maintained between 60% and 80%. Light conditions within the chamber fluctuated between 300 and 1200 μmol m^−2^ s^−1^. Treatment differences were determined by general linear model (GLM) repeated measures ANOVA.

Dry matter accumulation during the light period, standardized according to pod size, was not impacted by the water deficit treatment in pods that were undergoing expansion (Fig. 5, *P*=0.849). The amount of dry matter deposited in the pod generally increased by 7 and 15 mg g^−1^ over the 10 h light period for both well-watered and water deficit-treated plants.

**Fig. 6.**
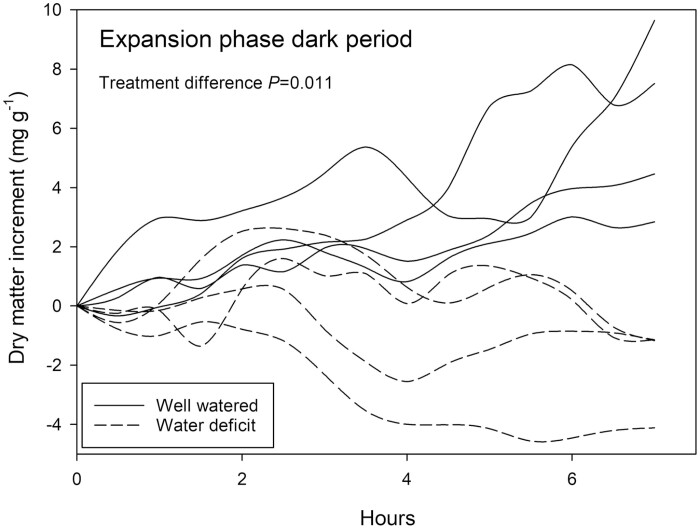
Relative change in solid matter (by weight) of *Phaseolus vulgaris* pod segments measured by NMRS for an 8 h period in the dark during the pod expansion phase. Plants watered daily to field capacity (solid lines) and plants supplied with 30% of calculated water use of control plants are represented (dashed lines). Greenhouse conditions were set at 25/15 °C with a relative humidity maintained between 60% and 80%. Light conditions within the chamber fluctuated between 300 and 1200 μmol m^−2^ s^−1^. Treatment differences were determined by GLM repeated measures ANOVA.

Dry matter accumulation in expanding pods differed between treatment groups during the dark period (Fig. 6, *P*=0.011). All water deficit-treated plants exhibited a loss of solid matter, whilst well-watered plants continued a net accumulation in the range of 2–8 mg g^−1^ over the 7.5 h period.

### Pod filling phase: water deficit elicits contrasting effects on pod growth during light/dark periods

**Fig. 7.**
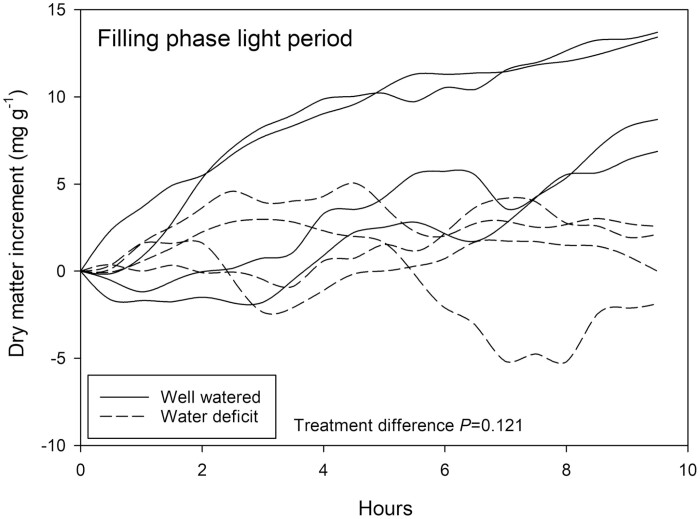
Relative change in solid matter (by weight) of *Phaseolus vulgaris* pod segments measured by NMRS for a 10 h period in the light during the pod filling phase. Plants watered daily to field capacity (solid lines) and plants supplied with 30% of calculated water use of control plants are represented (dashed lines). Greenhouse conditions were set at 25/15 °C with a relative humidity maintained between 60% and 80%. Light conditions within the chamber fluctuated between 300 and 1200 μmol m^−2^ s^−1^. Treatment differences were determined by GLM repeated measures ANOVA.

Relative rate of dry matter accumulation during the light period in well-watered plants was similar to that observed in the expansion phase of between 5 and 12 mg g^−1^ over the 10 h period (Fig. 7). In contrast, water deficit-treated plants exhibited net relative changes of between 2 and −2 mg g^−1^ dry matter. Statistically, treatment did not impact relative loading rate (*P*=0.121) seemingly due to the overlap between traces across the first 6 h period that was not present at the end of the dark period (Fig. 7).

**Fig. 8.**
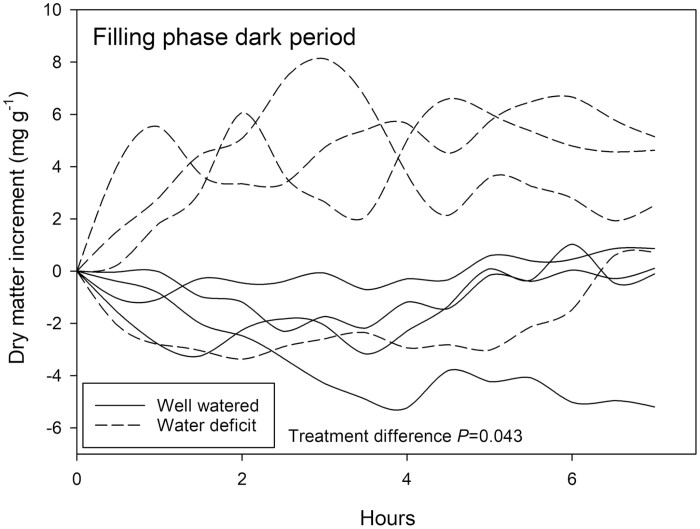
Relative change in solid matter (by weight) of *Phaseolus vulgaris* measured by NMRS for an 8 h period in the dark during the pod filling phase. Plants watered daily to field capacity (solid lines) and plants supplied with 30% of calculated water use of control plants are represented (dashed lines). Greenhouse conditions were set at 25/15 °C with a relative humidity maintained between 60% and 80%. Light conditions within the chamber fluctuated between 300 and 1200 μmol m^−2^ s^−1^. Treatment differences were determined by GLM repeated measures ANOVA.

Pod filling during the dark period was statistically larger in plants subjected to water deficit (*P=*0.043) than those that were well-watered, although absolute separation between treatments was not observed at the end of the treatment period (Fig. 8). For three out of four replicates in the water deficit-treated plants, increases in dry matter of around 4 mg g^−1^ occurred during the first 2 h of the designated dark period. A rapid reduction in dry matter detected between hours 3 and 4 of one of these replicates may be attributed to the pod moving within the coil. By the end of the dark period, water deficit-treated plants had increased between 0 and 5 mg g^−1^ dry weight whilst well-watered plants had decreased between 0 and 5 mg g^−1^ dry weight.

### Instantaneous pod dry matter acquisition does not correlate to whole plant photosynthetic rate

**Fig. 9.**
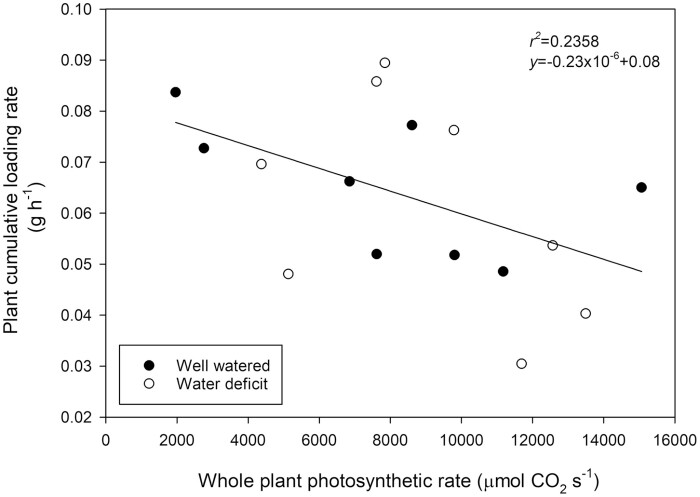
Calculated plant-scale pod filling of *Phaseolus vulgaris* measured by NMRS for an 8 h period in the light during the pod filling phase plotted against whole plant photosynthetic rate. Plants watered daily to field capacity (filled circles) and plants supplied with 30% of calculated water use of control plants (open circles). Greenhouse conditions were set at 25/15 °C with a relative humidity maintained between 60% and 80%. Light conditions within the chamber fluctuated between 300 and 1200 μmol m^−2^ s^−1^.

Calculated rates of pod filling at the plant scale during the light period did not scale with whole plant photosynthetic rate (Fig. 9). A weak, non-significant, negative regression was detected on pooled data of both water-deficit and well-watered treatments (Fig. 9).

## Discussion

Plants place great importance on the establishment and development of reproductive organs. Ensuring the completion of this process is of foremost priority to maximize fecundity and pass on genetic information to the following generation (see Nienhuis and Singh, 1988). From the perspective of the plant, the resilience of this process is critical and—at least at the scale of individual pods—takes precedence over maximizing seed number and ultimately crop yield (Hageman and Van Volkenburgh, 2021). The process of pod development is likely under developmental control, which in turn is strongly influenced by environmental cues (Braun *et al.*, 2014; Rolletschek *et al.*, 2015; White *et al.*, 2016). With the use of NMRSs to measure dry matter deposition in developing pods we have provided the first evidence for diurnal changes on loading rate, impacted by physiological status in response to environmental variation. This real-time investigation of loading rate has not only characterized variation in loading rate across the diel cycle (Fig. 2), but also represents the first *in situ* measure of pod ‘sink strength’ (for photoassimilates) during changes in resource availability. This enables the direct observation of realized pod development and loading under the influence of source-sink co-limitation (see for example White *et al.*, 2016).

### Short-term water limitation influenced whole plant photosynthetic rate but not pod number

We exposed *P. vulgaris* to a mild water deficit, inducing a significant reduction in net photosynthetic rate and stomatal conductance per unit leaf area (Table 1). Scaling of photosynthesis and stomatal conductance to the whole plant (Table 2) illustrated—at least in broad terms—a significant reduction in source strength for photoassimilates as no adjustment of leaf area was observed (Fig. 4). Importantly, whilst source strength was diminished, sink size (hence to some degree sink strength) was not reduced (Table 2). Reducing sink strength by the abortion of flowers and pods is a useful mechanism for plants to prioritize existing seeds in times of resource scarcity and has been seen as a problematic trait among many legume species under production (Beebe *et al.*, 2011). Pod abortion may be elicited by an array of resource shortages, the timing and severity of which may act singly or in concert (Leport *et al.*, 2006; Fang *et al.*, 2010). Conversely, the stability of pod and seed development necessitates a gradual independence from the physiological status of the plant, as reproductive viability of the developing tissue is prioritized (see Nienhuis and Singh, 1988). It is likely that the observed reduction in *A* and *g*_s_ over the course of the measurement period did impart a reduction of source strength on the development of individual pods, but some pods may have passed a threshold over which they are sensitive to such cues for impaired development. The level of water deficit used in the present study did not elicit pod abortion, but did lie within the scope of stresses that *Phaseolus* plants may encounter under field conditions in many parts of the globe. The application of this drought treatment therefore provides a viable platform to investigate the impact of water deficit and source strength on the processes of pod development.

The efficiency by which carbon was assimilated per unit of water used did not differ between treatments, indicating that *A* and *g*_s_ scaled accordingly. Equally, carbon isotope abundances commonly used for predictions of water use efficiency (Farquhar *et al.*, 1982; Farquhar and Richards, 1984; Seibt *et al.*, 2008) did not differ among treatment groups. These results suggest that *P. vulgaris* under the conditions experienced in this study exhibited moderate anisohydric behaviour (Tardieu and Simonneau, 1998). Combined, these results illustrate that *P. vulgaris* may adjust photosynthetic rates under medium term water limitation, but this adjustment was not of a magnitude to significantly influence the total yield per plant on a dry weight basis. This suggests that *P. vulgaris* exhibits higher photosynthetic rates than are needed to supply developing pods, both under well-watered conditions and under water deficit as imposed in the current study, and/or that significant ‘buffering’ of photoassimilates is achieved between carboxylation and the loading of dry matter into the developing pod.

### Pod development during the expansion phase

Monitoring of pod growth by NMRSs during the expansion phase of development revealed remarkably consistent increases in pod dry weight. The relative increase in proton density (relative to the proton density of the pod segment at the beginning of the measurement period) provides a standardized method that enables the comparison of loading rates among beans of differing size. Integrated measures of dry weight increase of a pod segment over a 10 h period illustrate the strength of repeated, non-invasive measures. The data presented here represent, for the first time, incremental gains in pod dry weight, measured several times within the hourly cycle for pod segments with between 0.02 and 0.12 g total dry weight. Further improvements in the sensitivity and precision of these measurements will be made through further developments in NMRS design and improved shielding from background RF noise (see for example Windt *et al.*, 2021). Relative loading rate of individual expanding pods was not impacted by water deficit in the light period during the expansion phase (Fig. 5) suggesting that (as noted earlier), photosynthetic rates appeared in excess of that required for growth and maintenance. In the light, sufficient capacity in photosynthetic production (for review see Zhu *et al.*, 2010) may be present to overcome the limitations imparted by the water deficit. Also, pod expansion (accommodating potential increases in seed number and/or size) may be semi-autonomous from the physiological status of the plant (Westgate and Grant, 1989). Relationships between the severity of stress and the thresholds at which that severity will impact on pod development may shed light on the processes that determine pod abortion (see for example Beebe *et al.*, 2013).

In contrast to the light period, relative pod loading rate in the dark period during the expansion phase was impacted and lowered by water deficit (Fig. 6). This result is surprising on the basis that pod expansion is driven, at least in part, by turgor pressure in the developing tissues (see for example Tardieu *et al.*, 2011). Water deficit experienced during this process thus might be expected to impact pod expansion more during the light period, when evaporative demand is high, than during the night period when plant and pod can replenish water stores. Why then is dry matter accumulation in expanding pods impacted during the dark period, but not during the light? Two hypotheses may be considered to explain the phenomenon. First, during the expansion phase the pods are growing rapidly, in girth but even more so in length. It is conceivable that under water deficit pods lengthen preferentially at night. During the day the pods will need to osmotically adjust and accumulate soluble sugars, but may have difficulty taking up sufficient water for expansion growth. This deficit may be compensated at night, taking up more water but less photosynthates. Sugars already imported into the pod and seeds may be respired and/or converted into more chemically reduced forms, driving pod expansion and growth. Due to preferential night-time expansion growth, the amount of dry matter in the field of view of the NMRS (the central 20 mm of the pod) may remain constant or be reduced, even while total fresh weight of the pod is increasing (see Fig. 2). The idea that developing pods accumulate sugars during the day to osmotically adjust would be in keeping with the observation that pod and seed water status is maintained at a high level, irrespective of the water status of the vegetative part of the plant (Patrick, 1994; Westgate and Grant, 1989; Patrick and Stoddard, 2010)

An alternative explanation may lie in the buffering of photoassimilate supply over the diel period. It is well known that leaves accumulate starch during the light period to facilitate photoassimilate supply during the dark periods (see Stitt and Quick, 1989; Sulpice *et al.*, 2009; Stitt *et al.*, 2010). For the present study, the realized impact on pod growth, exhibited only during the dark period, suggests that photoassimilate translocation into the pod is limited during this time. This may be attributable to a restricted ability to accumulate starch during the day period as a result of reduced photosynthetic activity, or to the need to osmotically adjust and maintain high concentrations of sugars throughout the plant. Observations at the plant scale (Fig. 9), however, suggest that photosynthetic activity is in excess of what is needed. Prioritization of growth, maintenance, and osmoregulation during the light period may impede the plant’s ability to establish sufficient starch or phloem-located reserves to sustain pod growth during the dark period. Exhaustion of buffering mechanisms, such as starch and non-structural sugars that mitigate short-term restrictions in photoassimilate production, may be an early indication of resource limitation and may prove useful in efforts to increase the resilience of plant function in response to short term stress conditions.

### Pod development during the seed-filling phase

At the seed-filling phase, contrasting impacts of water deficit were again observed among light (Fig. 7) and dark (Fig. 8) periods, but they were opposite to the pattern during the expansion phase. During the light period, pod growth was reduced in water deficit-treated plants with low or negative dry matter accumulation rates. In contrast, during the dark period, pod growth was higher in the water deficit-treated plants, particularly in the first hours of the period. The most important differences between seed loading in the expansion and seed filling stage in legumes are in the mechanism of phloem unloading. During seed formation, all photosynthates are translocated into the pod by means of the phloem. Before these sugars can be taken up by the developing seeds, they need to be released by the maternal seed coat into the seed apoplasmic space. During the expansion phase, unloading of sugars from the phloem still occurs passively (Milne *et al.*, 2018). Transitioning to the filling phase, however, the sucrose concentrations in the seed apoplasmic space have been found to strongly increase (Patrick and Stoddard, 2010) The high sucrose content appears to have a signal function, subsequently promoting starch accumulation in the seed (Weber *et al.*, 2005). Active (sucrose/proton antiport) phloem unloading facilitates sucrose accumulation against the steep sucrose gradient, and allows the build-up of a sucrose gradient into deeper layers of the seed (Borisjuk *et al.*, 2002; Milne *et al.*, 2018). Older studies suggest that pod loading in legumes is highly turgor sensitive (Wolswinkel and Ammerlaan, 1984; Patrick, 1994). We hypothesize that the daytime reduction in pod loading, and the night-time resumption of it, may be due to the strong daytime reduction in water potential and turgor of the water deficit-treated plants. Under these conditions postponing of seed loading until the dark period may be energetically advantageous. In addition, in the first hours of darkness the plant may be able to quickly translocate the pool of sugars that during the day were mobilized in the course of osmoregulation. The latter may explain the pattern observed in the first 2 h of the dark period.

### Whole plant pod growth does not correlate with short-term photosynthetic rate

The relative magnitude of pod growth (as a percentage of initial size) was similar between pod expansion and seed filling phases under well-watered conditions. This suggests that the absolute rate of dry matter accumulation under consistent conditions is a consequence of pod size. Consequently, we were able to calculate whole plant pod loading rate via the relationship between relative pod size and the corresponding loading rate. On this basis, pod loading rate did not correspond to photosynthetic rate at the plant scale (Fig. 9), highlighting the importance of alternative processes governing biomass partitioning that determine the source strength for photoassimilates to a developing pod. Combined with the observation that under ideal conditions photosynthetic rates appear to exceed the requirements for pod development, it would appear that increases in maximum photosynthetic rates and radiation use efficiency, as were proposed by numerous authors to improve yield (Zhu *et al.*, 2010; Kromdijk *et al.*, 2016; Kromdijk and Long, 2016), may have a less beneficial influence than expected (see also Evans, 1997; Smith *et al.*, 2018). In light of patterns observed between light and dark periods during seed filling and the decoupled relationship between photosynthetic rate and pod loading, the availability of water may provide more influence over pod loading and yield development rates than previously thought. The influence of water status on yield development has been highlighted for *P. vulgaris* (Broughton *et al.*, 2003; Beebe *et al.*, 2008; Cuellar-Ortiz *et al.*, 2008; Polania *et al*., 2016*a*, *b*) and more generally for production systems (Blum *et al.*, 1996; Chaves *et al.*, 2003; Chaves and Oliveira, 2004; Passioura, 2007; Chaves and Davies, 2010). Crucially, efforts to increase yield production via increased photosynthetic activity and radiation use efficiency may exacerbate water deficit through the expansion of photosynthetic tissues and therefore reduce the resilience of yield production. The manipulation of the whole plant balance between photoassimilate supply and water use (including the potential for artificial manipulation of leaf area closer to harvest) may provide useful tools in improving the resilience of yield production.

### Implications for the study of source–sink dynamics

The re-emergence of literature recognizing the importance of source–sink dynamics and its corresponding impact on growth have been the subject of extensive reviews (see for example Körner, 2015; White *et al.*, 2016; Smith *et al.*, 2018). Co-limitation of growth rate by both source and sink activity has substantial experimental evidence (see White *et al.*, 2016), despite the absence of real-time measurements of sink strength. For pod formation, developmental processes influenced by environmental conditions combine to produce complex circumstances that realize growth. A commonly accepted definition of sink properties is that proposed by White *et al.* (2016), stating that sink strength is the net rate of uptake (mol s^−1^) for a particular resource and that:


$$\begin{array}{l} Sink\;strength\;=\;sink\;size\;\times \;sink\;activity \end{array}$$

where the sink size refers to the total biomass of sink tissue (g) and the sink activity is the specific uptake rate of the resource (mol g^−1^ s^−1^). For a developing pod and its function as a sink for photoassimilates, the practical application of this definition presents several challenges. Firstly, non-invasive measurements of sink size are difficult without excision of the tissue, at which time no further measurements of ‘activity’ can be made. Second, approximating the rate of resource input into the sink has previously required specialized techniques such as short-lived radiolabelling (Minchin and Thorpe, 2003; Furbank *et al.*, 2004; Cuellar-Ortiz *et al.*, 2008; Roeb *et al.*, 2009; Metzner *et al.*, 2010; Thorpe *et al.*, 2011; Coello and Martínez-Barajas, 2016) that are low throughput and expensive. Here, we have developed a methodology based upon recent advances in technology that is standardized for both relative growth rate and developmental stage, and that can be applied across a short time course to infer comparative rates of yield development, hence sink strength with an important caveat.

For developing pods acting as a sink for photoassimilates, the quantity of sink activity has two dominating components—those of tissue growth and tissue maintenance. Consumption of photoassimilates by respiratory processes contributes to pod sink strength and is necessary even in the absence of incremental growth. Similarly, large pods may have low demand for respiratory substrate whilst small pods may have high demand as a consequence of high metabolic activity to fuel growth and expansion. It has been shown that respired CO_2_ from pods is almost entirely refixed by the components of the pod wall in *Cicer arietinum* (Furbank *et al.*, 2004). It is possible, therefore, that a similar process is exhibited in *P. vulgaris*, albeit a distant relative within the legume family. Consequently, incremental growth for the present study can be used as an approximation of sink strength for photoassimilate supply.

Transport rate into the developing sink is the sole indicator of sink strength with the caveat that the absolute quantum is only relevant under the conditions in which that rate is measured (i.e. a given source strength) as per the assumptions of co-limitation. The application of NMRSs to monitor absolute growth rates of *P. vulgaris* pods has for the first time enabled the direct measurement of net sink growth (hence realized sink growth) in real time. Under the conditions of this study, this non-invasive, continuous measurement enabled the calculation of time-integrated realized sink strength and tissue growth showing the independence of sink strength from that of the source. The practical applications of this technology are manifold. Firstly, fundamental questions regarding source–sink co-limitation of yield development can now be investigated with a unique quantification of sink strength. Second, the development of cost effective technology enables the extrapolation of fundamental research findings to rapid and repeatable screening tests for a range of traits influenced by source–sink dynamics.

## Conclusions

*In situ* measures of pod development have uncovered several processes of yield development that were previously uncharacterized. Here we have shown that transfer of dry matter into the developing pod is impacted by resource limitation (water) over the diel cycle and that the dynamics of this relationship differ between light and dark periods. The magnitude and nature of this influence vary, at times counter-intuitively, highlighting the buffering capacity for photoassimilate supply to a developing pod. From a plant perspective, decoupling of pod development from plant physiological status is a logical approach to maximize fecundity and reproduction. Using the NMRS to quantify the dynamics of this relationship and how it varies among genotypes is likely to be useful in efforts to maximize yield production.

## Data Availability

Supporting data for this study can be provided on request.

## Abbreviations

FID: free induction decay
CPMG: Carr–Purcell–Meiboom–Gill
MRI: magnetic resonance imaging
NMR: nuclear magnetic resonance
NMRS: nuclear magnetic resonance sensor
PD: proton density
RF: radio frequency
